# Thematic analysis of stakeholder perceptions for co-creative healthcare XR resource design and development; traversing a minefield of opportunities

**DOI:** 10.3389/fdgth.2024.1341349

**Published:** 2024-04-10

**Authors:** Panagiotis Evaggelos Antoniou, Annita Varella, James D. Pickering, Charalambos Chatzimallis, Vassiliki Moumtzi, Panagiotis D. Bamidis

**Affiliations:** ^1^ Laboratory of Medical Physics and Digital Innovation, School of Medicine, Faculty of Health Sciences, Aristotle University of Thessaloniki, Thessaloniki, Greece; ^2^ Leeds Institute of Medical Education, School of Medicine, Faculty of Medicine and Health, University of Leeds, Leeds, United Kingdom; ^3^ ViLabs (CY) LTD, Limassol, Cyprus

**Keywords:** XR educational resources, virtual reality, augmented reality, co-creation, Agile methodology

## Abstract

**Introduction:**

The expansive curricular volume of healthcare education makes a necessity the incorporation of innovative methods and immersive media in it. The core challenge in such approaches is the timely development of relevant immersive content such as Virtual, Augmented or Mixed Reality (VR/AR/MR) resources for healthcare topics. There is currently significant interest in the use of co-creative methods for streamlining immersive content development.

**Methods:**

A core research pursuit in this translational research field is the formulation of evidence-based, optimized workflows that streamline immersive content creation allowing for rapid expansion of innovative educational approaches in healthcare curricula. The purpose of this paper is to aggregate the perceptions of healthcare technologists and educators who participated in a series of co-creation sessions in order to elicit their best practice insights for design and development of XR educational resources using co-creative methods.

**Results:**

According to our thematic analysis, findings of the qualitative study demonstrated that a rigorous organizational approach is required to maintain a constructive exchange of information and to keep the design process alive for both content and technical experts. In addition, rapid prototype and display of co-created features can empower their contributions and help them design more efficiently.

**Discussion:**

Co-creative content production can benefit from adaption of existing frameworks and lightweight authoring environments that can facilitate generalized XR content development use cases.

## 1 Introduction

In the 20th century, the quantity of instructional material that healthcare practitioners must master has skyrocketed. Critical medical knowledge necessitates an adequate theoretical foundation in addition to implicit knowledge and experience (1). To this aim, the need for innovative educational approaches and practices is required. Immersive experiential technology can support medical education to flourish in this environment. Through their enhanced engagement, virtual patients, chatbots, and Virtual, Augmented, or Mixed Reality (VR/AR/MR) applications have a significant impact on the affective and educational condition of healthcare students (1–4).

Key obstacles in providing materials and innovative educational activities are time and cost overheads. A proposed solution for timely creation of relevant and valid immersive content is the implementation of participatory design methods. Co-creation as a healthcare educational digital content design methodology, is only lately gaining traction. With some conceptual works such as the ASPIRE framework (5) initial observations indicate that co-creation is a feasible method for streamlining XR content creation and reducing overheads (6). Participatory design methods have also been marked rather early on as plausible knowledge transfer methodologies (7–9). This work aims, through a thematic analysis of stakeholder interviews, to elicit optimal processes and workflows for co-creative design of XR resources. The end goal of this process is the reduction of development time for healthcare XR resources, the minimization of technical capacities required by a healthcare technology unit and the smooth transfer of educator requirements to technical specifications and implementations. To contextualize these aims, a brief discourse of the state of play in healthcare VR and participatory design methods follows.

### 1.1 Virtual reality in healthcare education

Extended Reality (XR), comprising AR, VR and MR, has enabled students to visualize abstract laws in physical ways (10–12). The sensory immediacy of these technologies results in an intuitive anchoring of essential information for the learner and facilitates paradigm building, which, in turn, enables learners to acquire robust and deep knowledge while minimizing the likelihood of establishing or maintaining conceptual errors (13).

The medical industry already possesses a body of immersive content. In 2019, the Royal College of Physicians acknowledged VR as a “…powerful educational tool for defined learning objectives…” (14) and listed multiple applications in medical education and surgery. In terms of resources, the global VR market was $3.10 billion in 2019 and is projected to reach $57.55 billion by 2027 (15, 16). This example demonstrates the possibilities for immersive content development in healthcare education. Nonetheless, this necessitates the comprehensive design and development of such resources in order to first test them and then implement them in practice. According to a previous study, the cost of planning and implementing VR training exercises for hospital-based healthcare practitioners was around $106,387.00 (17). Considering the reusability of VR resources, this was subsequently deemed a viable approach. However, the ongoing evolution of medical knowledge and training can quickly render VR resources unusable, hence diminishing their reusability. Therefore, it is essential to lower the implementation and design expenses of VR resources and to distribute the development burden of such resources. This is where participatory design methods and co-creation approaches can be utilized to address the issue of content generation and availability.

### 1.2 Co-design and co-creation process

Participatory Design (PD) is regarded as one of the most crucial prerequisites for an acceptable and effective design and development process of reusable e-resources, as it relies on the early and active engagement of all key stakeholders (18). PD techniques promote and strengthen collaboration among team members and permit developers and educators to evoke collective creativity throughout the design and production of educational resources (19). Particularly, PD approaches include regular meetings with end-users, clinical and technical specialists, in real time, or focus groups and surveys with the primary objective of eliciting a list of user and technical requirements. This can be accomplished through co-creation workshops that increase the early active participation of stakeholders as co-creators and the rapid prototyping capability of the technique (20). Specifically, stakeholders are asked to construct use case scenarios in the early stages of the project in order to elicit a well-defined set of needs. Consequently, a hybrid strategy incorporating design thinking approaches can be utilized. This method permits the investigation of the stakeholders’ true demands and the elicitation of design concepts that encourage valuable and immersive results (21). The working prototype of the produced resources is routinely displayed by the developers to the stakeholders during co-creation workshops in order to collect feedback, recalibrate, and proceed with the necessary modifications (22).

Co-creation provides dynamic support for the resource design and development process and is characterized by a stakeholder association that reflects mutual physical, mental, and business practices, as well as the ability to split the creative process (23, 24). This process fully engages stakeholders in the co-design process (25, 26) underlining the significance of mutuality, receptivity, and non-hierarchical relationships (27, 28) as a co-design characteristic. This level of insight into the co-creation process enables the identification of (7) information distribution as one of the most important co-creation effectiveness drivers. It has been suggested that working with diverse stakeholders in a co-design setting facilitates the production of more inventive concepts and ideas (8). During the co-design process, stakeholders, for whom the final resources are being built, are the experts and so have the opportunity to contribute to the development of information, ideas, and concepts (9). Throughout the whole design and development process, they are able to provide the required tools for the expression and ideation of their requirements, as well as make crucial judgments (9). Co-design is employed as a technique for collaborative design, with key stakeholders collaborating concurrently in the co-creation process. Co-creation is predicated on the concept that the participation of end users is crucial to the creative process, bringing together each other's perspectives, beliefs, wants, and preferences and jointly producing solutions (29).

The adoption of co-creative methods in the production of immersive content has received considerable attention (30). This methodology's central concept is the flexible scheduling of combined educator-technologist activity. This can be accomplished through Scrum pushes encouraged by the Agile development paradigm (31), utilizing semantic back-ends in pervasive game development platforms. However, the effective deployment of co-creative methodologies demands the precise specification of the objectives and goals of the immersive content, i.e., Agile requirements elicitation. In addition, the co-creation and co-design workshops for XR healthcare materials must be refocused in light of contemporary research in the relevant sector.

Having conducted a series of several participatory workshops between technologists and healthcare education experts for developing XR resources for the ENTICE project (https://entice.eu/), this qualitative study aims to outline what works and what doesn't in participatory methods during the development of XR healthcare resources. Specifically, in that context, this work has the overall aim of exploring the core needs for successful co-design of XR resources for medical teaching. To achieve this overall aim we have set out to explore the following study objectives: (1) What are the core concerns of XR resource co-design teams for medical teaching? (2) What do medical education and technology stakeholders consider as key enablers for supporting co-design of medical XR resources and episodes?

## 2 Methodology

### 2.1 Co-creation methodology and participants

This study included eight co-creation participants and active contributors from the ENTICE resource co-design team. Academic and technological backgrounds, as well as levels of co-creation experience, ranged from highly experienced co-creative technologists and researchers to academics and coders with little prior experience. The backgrounds of the participants in this study reflect the evolution of the XR immersive educational materials and their interdisciplinary nature to some extent. Two (2) learning technologists (software engineers), one (1) technical manager, two (2) senior medical educators, and three (3) teaching assistants participated in the interviews. All participants were core members of the XR team from the early stages of the project up until the final developments and were actively participating in the co-creation sessions.

It must be noted that co-creation sessions took place both during and after the COVID-19 pandemic. As such some were conducted online through teleconferencing software and others were face to face meetings. Six online sessions took place during the pandemic and over 10 took place face-to-face after the restrictions for the pandemic were lifted. Digital brainstorming boards, specifically, miro (miro.com) was used for allowing the depiction of participants’ ideas. Each co-creation session lasted between 90 and 180 min. In several of these sessions demo builds of the resources in desktop or VR form were presented and the team brainstormed on them. All sessions were video recorded and these recordings were the source of the XR resources’ design documents. Regarding the interviews, a member of the research team personally interviewed each participant. A problem centered interview approach was followed to touch on a variety of aspects for the co-creation process.

### 2.2 Design and procedure; problem-centered interviews with developers and educators

To get the necessary information, problem-centered interviews, a technique commonly utilized in qualitative research, were conducted. 1 interviewer and 1 analyst (Author #2 and Author #1 respectively) formed the thematic analysis team. The most useful features of the interviews were their ability to illuminate the participants’ subjective experiences, viewpoints, and impressions of the educational resource design and development process. Problem-centered interviews are characterized by a conversational process that allows interviewees to articulate their thoughts and subjective experiences. This interviewing approach reflected the key components of qualitative research, such as openness, adaptability, and orientation. In accordance with these ideas, the interview guide was developed as an organized discourse rather than a hard questionnaire, with the goal of eliciting spontaneous comment on all main aspects and viewpoints of the co-creation process. Participants were specifically requested to express views about their co-creation experience that were not necessarily addressed by the interviewer's inquiries. However, in order to establish and preserve a structured conversational flow, spontaneous follow-up inquiries and brief comments were also provided. The interview guide, as previously stated (9), was organized around five major subject categories. It ensured that the participants’ collected experiences were comparable and complete.

The interviews were recorded and transcribed. Each member of the thematic analysis team created their own initial thematic analysis and then a common discussion merged these results into a common thematic list. Upon preliminary analysis of the transcripts several different themes diverging from the interview's prescribed thematic areas emerged. In light of that fact we pivoted to an ab-initio thematic analysis to explore these new emerging themes. Thematic analysis is a staple method of extracting meaning and organizing feedback from qualitative data. In a rather seminal paper describing the method in 1985, for healthcare Benner (32) cites Lazarus (33), when describing the main challenge of thematic analysis as being “the task (..) to uncover the meanings in everyday practice in such a way that they are not destroyed, distorted, decontextualized, trivialized, or sentimentalized”. Aronson (34) describes the specific methodology we followed in detail at a later work. Summarily, thematic analysis steps are: (a) Collect and transcribe data, (b) Identify all data that relate to already identified patterns, (c) Combine and catalogue related patterns into sub-themes, (d) Follow through on sub-themes, (e) Build arguments for choosing themes and sub-themes according to the literature and finally, (f) Formulate a story line that stands with merit and facilitates deeper comprehension of the process and its underlying factors. Following this approach other authors have refined and presented a more recent perspective on thematic analysis, Braun, and Clarke (35) report that thematic analysis is a method for identifying, analysing, and reporting themes within qualitative data. Themes are identified by the researchers as important about the data in relation to the research question and represent some level of patterned response or meaning within the data set. In their work Braun and Clarke, (35), essentially present the same process as Arronson. However the newer by Braun and Clarke focuses more on the process itself and offers a more contemporary view and a more streamlined workflow. Specifically, thematic analyses is defined as six phases: (a) Familiarizing yourself with your data, (b) Generating initial codes, (c) Searching for themes, (d) Reviewing themes, (e) Defining and naming themes, (f) Producing the report. This workflow was also followed in the present work. In the present study, verbal data retrieved from the interviews were initially transcribed into written form to construct a thematic landscape. Thereupon, the process involved the production and collation of initial codes annotated in the transcriptions from the data to organise them into meaningful groups, resulting in a list of different codes identified across the data set. This process allows for the codes to be grouped within different themes, which will later on be subdivided into subthemes. After reviewing the collated themes, data within themes appeared to be cohere together meaningfully, while clear and identifiable distinctions between themes were eventually conducted. Finally, themes were defined and refined by the research team, resulting in the final list of themes and subthemes.

## 3 Results

We have chosen to present our thematic analysis in a series of tables. Each table describes the identification of each major theme and its subthemes from participator feedback. In each table the first row presents a series of characteristic quotes that the researcher identified as dominant from the steps (a) to (d) of the process defined in the thematic analysis literature. These are not the only, verbatim available, quotes about a topic. They express, however, a significant portion of the participants’ feedback during the interview. In the next column we provide the researcher's rationale for their assignment of these quotes as reflections on a specific theme and subtheme, step (e) in the thematic analysis process. It must be noted that these assignments were based on the specific quotes, their contextualization in the text, as well as their purview on the whole interview of the participant. Next to it we present the themes and subthemes that were identified from these processes. We elected to present our data in tabular form in order to provide a more coherent and concise description of the themes that we identified throughout our analysis. As such, while a brief analysis write-up is presented in tabular form, we found that the diversity of the themes of our case would create an encyclopedic, disjointed analysis if presented in narrative form. It must be noted that thematic definitions in this analysis are highly contextualized. For example some subthemes are synonyms but differing in the context of overall theme. “Tools and media” is present as a subtheme both in the “Organizational rigor” theme and in the “Authentic communication” theme. In the first theme “Tools and media” refers to planning and project management tools while in the second it refers to teleconferencing tools. Such contextualization is important both for demonstrating the breadth of every identified major theme, as well as demonstrating the role of “enabling” subthemes in many identified major themes. Also, some major themes like can pivot as subthemes in a different context. That is the case of the major theme “Organizational Rigor” which contain as a subtheme the “Facilitating authentic communication” subtheme. It is clear that this subtheme is, as a term, similar to the major theme “Authentic communication”, but the real meaning of the two items is different in context. In the first case “facilitating authentic communication” refers to the organizational provisions needed to support the subtheme, while in the second case “Authentic communication” explores user feedback on a totally different conceptual pivot, that is the facets and needs of communication in co-creation process. In the context of these notes we present the following tables of specific major themes as they were identified in our analysis.

**Cross-disciplinary collaboration**, its limitations and benefits was a major theme that was discussed by all interviewees. Its subthemes and relevant quotes from the interviews are summarized in Table 1.

**Table 1:** Cross-disciplinary collaboration theme and subthemes as expressed by the participants.

Quotations from interviews	Definition & tefinement	Themes	Subthemes
*“It is important to get involved with the doctors, especially during the feedback elicitation. The developer would also need a direction from the beginning of the project. The development team is much needed to be involved in the beginning with the help of the medical expertise.”* *“It is a very effective way of conducting such methods, involving multidisciplinary people in the workshop.”*	Participants highlighted the importance of involving different actors from diverse backgrounds during the design and development phase of the XR resources.	Cross-disciplinary collaboration	Diverse participation
*“People didn’t have the necessary time to prepare for the detailed conversations we needed to do during the co-creation sessions, because time has been a luxury we haven’t had. It’s been a perfect storm, a lot of people have struggled to maintain projects during the last two years.”*	Time and stages of the actors’ involvement within the design and development phase of the resources was a recurring theme. Participants found it important to involve both content experts (doctors) and developers at early stages of the process, to identify real needs of the educators and allow them to provide essential feedback and knowledge on the resources. At the same time, the development team acquires the expertise to create the educators’ cohesive vision of the resources.		Timely involvement
*“The Medical personnel were involved in the early stages of the design and development phase. In case they did not, there would be a lot of revision if involved in later stages”*			
*“Important to involve actors from the early stages of the development as they are the target users, giving valuable feedback that enables the effective development.”*			
*“Involve doctors and developers at the early stages; you can have discussions about the context and take it from there in terms of designing. In a later stage there is something to see, there is already progress and have the opportunity to see the product. This two-stage approach followed was very beneficial”*			
*“Maintain the involvement of actors all stages of the process. Stakeholders were involved in the design, implementation providing back and forth feedback and deployment we will explore the feedback.”*			
*“Technology and pedagogy expertise somehow create a cohesive vision from the first sessions and then drive with this vision. Brainstorming on the first sessions and then drive through with a concise vision.”*			
*“I am not a medical student myself but during the co-creation session I learned a lot of things even from the students and I also feel like I have added my contribution. If each group was invited at different times, the interaction would have lacked knowledge exchange. Each discipline completed the other—it is important to have diversity. Everyone was sharing ideas; this could not have happened so effectively if everyone was from the same discipline.”*	Knowledge transfer was an aspect especially mentioned by the technical development team. Educators’ knowledge and experience transfer, a type of knowledge crosspollination was necessary in order to proceed with the resources’ design and development throughout the whole process.		
*“Very interesting sessions with experts from different backgrounds, it was fascinating the fact that one idea introduced the next one and then we were building above that. The results were very interesting. I could listen to different perspectives and aspects.”*	Participants mentioned Brainstorming as a need to be promoted within open discussions among different actors. Every actor should be able to express ideas and listen to different perspectives of each topic. This fertile ground for ideas was deemed necessary by the participants for facilitating cross-disciplinary collaboration.		
*“Resources could be exploited in blended learning, preparation of the students before the lesson or during class. It was interesting to identify the context of the exploitation of the VR.”*	From the interviews it became clear that educators acquired the capacity for identifying educational context for technological resources to be effectively exploited.		
*“The participation at a later stage was important since we had the opportunity to see what was created in the first sessions. Not everyone has experience on designing resources, so it was valuable to provide feedback at later stage with the students.”*	Across interviews access on varying stages of resource development was deemed important. Also opportunities for feedback in each iteration was considered valuable contribution of the co-creation process to the development of the resource.		
*“Many times, the early point of entry related to lack of design focus in the sense of having many ideas and not a unique vision. We had multiple ideas but slowed down the design process as there had to be a filtering stage, in order to collect and create a summary into a concise design vision. That was one of the lessons. We had several sessions without creating an initial vision. This might have caused a delay.”*	Participants, especially educators outlined the need for clarity of purpose throughout the design process. This pertained, mainly, to the risk of technical development producing results that were impressive but detracting from the educational goals. Participants emphasized the need for recorded specific educational objectives, clearly linked to technical outcomes, in order to maintain a coherent vision throughout development.		

**Organizational rigor,** the needs for appropriate collaborative storyboarding tools and well facilitate workflows, was also extensively mentioned in the interviews. Specific sub-themes with relevant quotes are summarized in Table 2.

**Table 2:** Organization rigor theme and subthemes as expressed by the participants.

Quotations from interviews	Definition & refinement	Themes	Subthemes
*“It was a free experience—everyone could share thoughts and the whiteboard helped, the format was well organized”* *“Means used were good because they made the workshop more interactive. It was important to have a face-to-face session and have everyone sit around and capture and put down the thoughts of everyone in a concrete way.”* *“Liked the flow of the workshop—I liked our discussion, but I cannot remember how useful it was to have the board. Documentation is important, so I guess it was important for this purpose. Maybe a more systematic involvement of the board would be useful. MIRO could also be used.”* *“Materials were appropriate, the design tools and collaboration methods especially during the pandemic were adequate. Core suggestion would be (impossible due to covid), co-creation shines when face-to-face in the same room would be ideal to promote design thinking.”*	Participants stressed the need for the iterative process to be implemented within organized sessions among all relevant actors. They emphasized that need for digital or traditional means of collaborative working to facilitate the flow of the conversations and maintain appropriate records and documents. Tools and means, digital or physical are necessary to promote and enhance collaboration and support design thinking procedures.	Organizational rigor	Tools and media
*“The drive and vision of design passed through my coordination. Lessons learnt—initiate with brainstorming session, as we did in the project, and then design the summary of the sessions possibly after the first session, work with these and towards these through building specific and consistent building blocks from the contributors.”*	The need for new digital resource iterations in each workshop, if possible, was emphasized. Extremely detailed planning each step of the design and development is necessary to ensure the quick iterative cycles and consistently build blocks of work towards the final phase.		Need for extremely detailed planning for quick turn-around cycles
*“Co-creation is very similar and essentially the same with Agile. The difference is that you have diverse audience. You have iterations. A more closed fusion of scrum and co-creative approaches could be devised—have a product owner team that follows all scrum procedures but not as a decision by committing but as cohesive group that creates specific points after each co-creation meeting”*	The various roles of the participants need to cohesively come together in the co-creation procedures. Authentic, seamless communication was deemed the key factor to initially generate ideas and later facilitate productive and to-the-point discussions.		

**Authentic communication**, the need for it, necessary pre-requisites and limitations stemming from inherent cross-disciplinary barriers were touched and expanded upon as summarized in Table 3.

**Table 3:** Authentic communication theme and subthemes as expressed by the participants.

Quotations from interviews	Definition & refinement	Themes	Subthemes
*“Shortage of time, wanted to talk more about the research project and the overall endeavor”*	Brainstorming is sometimes chaotic in its creativity. The need for more time in-session for discussion clarification and refocusing of ideas was apparent from participant feedback	Authentic communication	Time to participate
*“There were pauses between questions so there was space to make any comments and express ideas.”* *“It was fine, everyone had different type of way to communicate and had different roles within the workshop, and everyone gave space for each other.”* *“Really productive discussion and everyone was equally involved. I felt comfortable to share my ideas.”*	All participants agreed that participatory workshops should be an open forum where discussions can elaborate on productively communicating regarding the topic of interest. While structured the need for free interactions better fit the purpose of the overall brainstorming discussions.		Open forum
*“Much better to be in person, there is a much freer communication and easier to immerse yourself into the project and into what other people are saying, really liked the affect that everyone could try the VR. It was a multifaceted experience.”* *“Communication was effective, and facilitators could assist the process. The face-to-face interaction was important, we could constantly ask questions and it worked fine.”*	With the pandemic and the constant teleconferencing happening in every aspect of work and leisure, participants Face to face interactions when re-allowed led all participants to feel far more expressive and productive.		Face2face need
*“Materials were appropriate, the design tools and collaboration methods especially during the pandemic were adequate. Core suggestion would be (impossible due to Covid-19), co-creation shines when face-to-face in the same room would be ideal to promote design thinking.”*	While the need of face to face meetings was ever-present the Covid-19 pandemic necessitated the use of digital tools and media for maintaining collaboration during the pandemic.		Tools and media
*“Facilitators had excellent peoples’ skills which are required in such sessions. Sense of initiative and leadership is important in order to drive the conversation constructively and without wasting time. Conciseness, peoples’ skills, leadership skills.”* *“Well-prepared, obvious they had experience in the field. Room for improvement, it would be important to have some videos or tutorial on how to experience the VR so that we save time.”*	Especially in the environment of remote collaboration (but also during face to face sessions) it was apparent that facilitators should demonstrate excellent communication skills along with being well-prepared for the conduction of the co-creation sessions.		Need for appropriate facilitation
*“Decision making within the co-creation process the issue is that in every group there are leaders, silent people, and followers. It is uncertain, on whether we could bring forward the more silent people. However, since we usually had one pedagogy expert and several development experts, maybe this is not applicable here. The opinions of the educators were always taken into consideration but there was minimal interaction and input of the developers. This created disparity on the expectation for each participant. Maybe a more cohesive group that didn’t have managers along with the technicians would be a better idea.”*	Participants with different roles offer diverse insights within the co-creation process. A diverse group is important in order to provide a diverse set of mindsets and voices, leading to effective product design. Care of group composition is essential in order to avoid either technologists dominance in implementation or educators feature creep that cannot be implemented or is necessary.		Through care of group composition

**Authentic stakeholder engagement** and its prerequisites were discussed and are presented in Table 4.

**Table 4:** Authentic stakeholder engagement theme and subthemes as expressed by the participants.

Quotations from interviews	Definition & refinement	Themes	Subthemes
*“Preparation before workshop is not needed. Maybe we would have created specific expectations and be biased before the session.”*	Participants recognized that coming “trained” with preconceptions about their peers’ roles will not effectively contribute. Educators who try to think like developers, or developers who try to wear the hat of content expert limit the capacity of each stakeholder for contribution. A clean slate is needed for authentic engagement of each stakeholder.	Authentic stakeholder engagement	Clean state
*“Would like to get involved in similar projects in the future. The VR could definitely be implemented within medical practice in the future, very useful especially for Neuroanatomy.”* *“A learning experience. VR could be used as a solution of learning and education within Universities. I would need such a way to visualize things in an interactive way like the VR.”* *“Medical students were participating, they reflected on the user needs and this will assist in meeting the challenge in medical education.”* *“The practice needs come from the pedagogy experts. The core of design was based around what they required and what the technical team strived to achieve to get as close as possible to their vision of the resource. Better evaluation of this can come from the actual evaluation of the VR. We got a close as possible to the original vision of the pedagogy experts and as such it should reflect a lot on what the practice requires.”*	A strong need for future endeavors on medical education and, specifically, on the development of VR educational resources was highly reported. All stakeholders seemed to be truly interested in the VR resources and felt that there is genuine need of a “product” that caters to these needs. They could all envision the exploitation of the VR resources within the medical curricula.		Need of product

Finally, **educational rigor** and the role of content co-creation as an educational method was brought up and is summarized in Table 5.

**Table 5:** Educational rigor theme and subthemes as expressed by the participants.

Quotations from interviews	Definition & refinement	Theme	Subthemes
*“Very educational experience for both sides (experts and students)”* *“Covered most important aspects in terms of clinical practice”*	It was interesting to note that several of the participants observed that the co-creation process provided genuine education for them. Most of them implicitly identified the potential of the co-creative process as an authentic educational process.	Educational rigor	Educational potential of co-creation

## 4 Discussion

**Figure 1:**
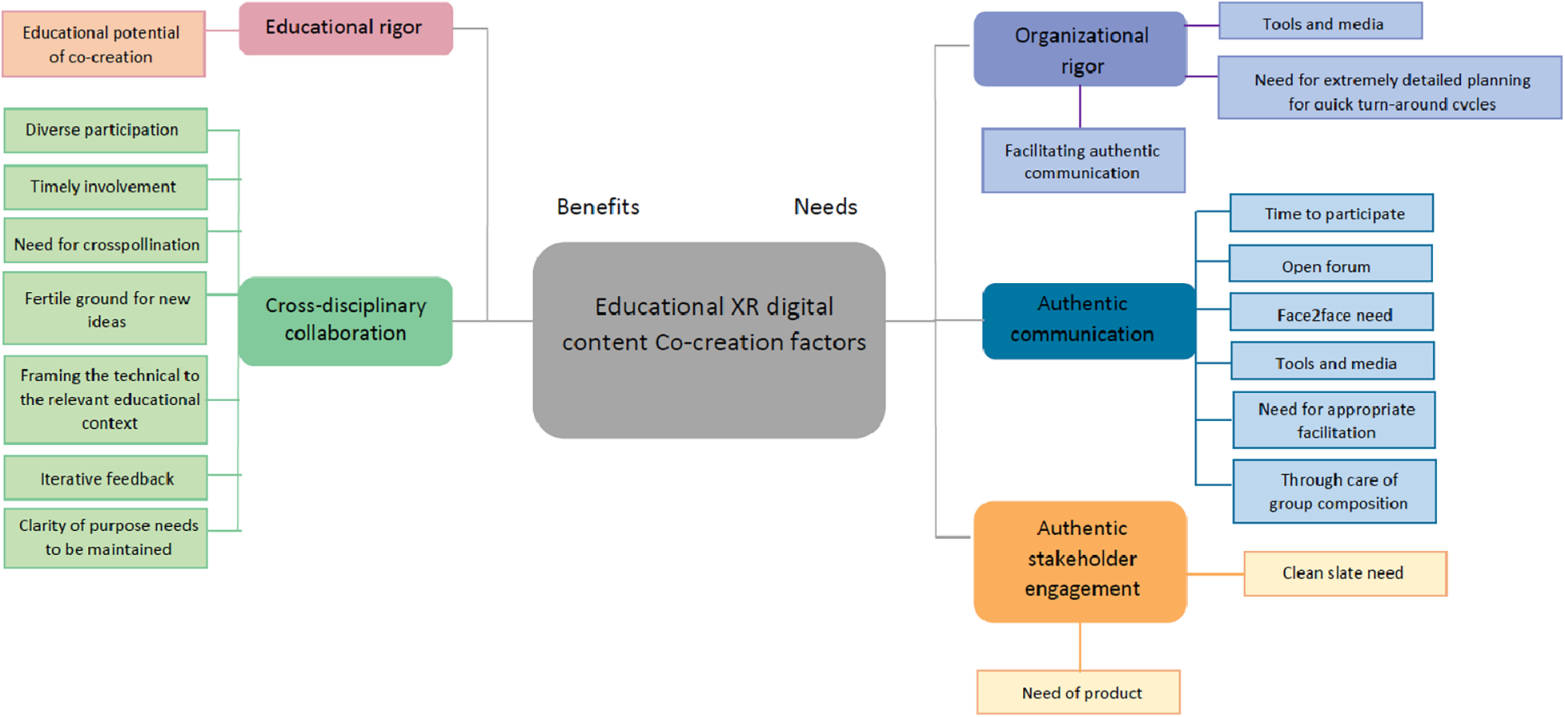
Mind-map of themes for educational XR digital content co-creation as they emerged from participants’ interviews.

This work pivoted off a previous work (6) that explored existing themes and pitfalls in digital content co-creation. Expanding the interviewee base, this time we attempted a new, bespoke, thematic analysis in order to explore new emerging themes that were only hinted upon in the previous work but were expanded upon in this study with more participants. Bringing together these themes and topics we conceptually summarize them in the mind map of Figure 1.

The key benefit from the co-creative approach in XR digital content design is the facilitation and expansion of **cross-disciplinary collaboration**. A key gap in digital content creation is that between the technical implementation team and the content experts. Alleviating this gap through timely and contextualized cross-pollination between these essential stakeholders is the main benefit identified by the participants of this study. For that, they identified the three (3) needs: (a) demonstrable clarity of purpose at all times of the co-creation process; (b) timely involvement of all stakeholders and (c) continuous iterative feedback. In essence, the participants emphasized the need for continuous, consistent involvement of the content experts with incremental, ever evolving iterations of the developing resources.

A similar outcome emerged from the themes that related to the needs for effective and efficient co-creation of XR digital content. **Authentic stakeholder engagement** emphasized also the need for a “clean slate” of technical capacities and the need for a “product” after every discussion to iterate upon. Also, following the previous needs, **organizational rigor** thematically emphasized on providing a very robust toolset and supporting media to enable the content experts in conveying their ideas into implementable features for the technical team.

In itself, the theme of **authentic communication** was expanded upon by several of the participants. To enable it, several participants emphasized on authentic presence, through face to face, instead of remote collaboration. Openness and appropriate, well defined facilitation was again brought up as key enablers for authenticity in communication. Compounding on these, participants mentioned the need for ample time of participation and balanced composition of the co-creation participants’ groups in order to not degenerate the session into either a developer sprint or to a lesson planning session.

An interesting note of one participant was the educational potential of the co-creation approach. As they observed, during the participatory design session, exchange of knowledge took place and some subject matter knowledge was transferred to members of the technical team. It is apparent that the **educational rigor** of co-creative methodologies is undocumented and such an anecdotal mention is of no real, evidence based, consequence. However the potential of co-creation methods for cross-pollinating knowledge transfer is documented (7–9).

From this thematic analysis two key requirements emerge for successful and efficient XR content co-creation in healthcare education.

1. Rigorous organizational process. Co-creation sessions present a warm and loose discussion based front. However, in order to be productive, facilitators need to have very structured discussion plan and collaboration tools (e.g., whiteboards, sticky notes and other enablers) to maintain a constructive exchange of information and keep the design process active for both the content and the technical experts.
2. Rapid prototyping and demonstration of co-created features. The key enabler for a successful co-creation session is a demo of the features that have been previously discussed. A common truism amongst all participants in our XR content co-creation sessions was «if you don't have a new prototype, let's not meet”. Through hands on experience the content experts can further enable their visions and understand, tacitly, technical limitations. In an ideal situation, a content authoring system that could implement in real-time rough ideas and storyboards might enable even shorter development cycles.

From these results it became clear that the themes of cross-discipline collaboration and organizational rigor have been emphasized. This is unsurprising, since these are the core hurdle in resource production and in bringing the educators’ needs exactly into the technical development pipeline. While rigorous topic presentation, meeting presence and basic communication strategies are always important, the core enabler for effective co-creation is authentic collaboration. In that context, on one hand, cross-discipline communication is the key enabler for this collaboration. On the other hand, organizational rigor provides the real world facilitation that allows these diverse groups to communicate effortlessly.

## 5 Conclusions

In earlier research (30), four crucial steps to the co-creative process in XR resources were discovered. Planning and preparation, actual co-creation, technical facilitation and training, and rapid prototyping comprise these steps. The “virtuous cycle” that enables quick deployment of XR resources and encourages simplicity of design and development across themes or even educational institutions uses this method to create an ever-improving fresh jumping point of feedback. Participatory design has therefore evolved as a means of democratizing digital citizenship, made even more important in light of the current pandemic (36). Democratization of XR content development in healthcare education may be possible by combining these procedures with Agile approaches. This qualitative methods study, aimed to cover two needs. First, it provided a set of practical considerations for organizing effective and efficient XR content co-creation sessions. Second, it points to the steps required for facilitating easier participatory creation for XR healthcare educational content.

To put this work in perspective, it is important, beyond its results to outline the core limitations that we identified and aim to alleviate in future research. The first and more important limitation is that of scale. This was a thematic analysis study that was conducted with only one team. That was a diverse multinational and experience team, however the fact remains that a thematic analysis on a cluster of such teams would hold further merit and is something that we are aiming to pursue in future work. Secondly, the educational scope of the team was medical teaching. All educators were medical doctors and anatomists working in medical schools. As such generalizing for the co-creative pipelines for other healthcare professions is difficult. Replication with more diverse groups of educators can help widening the scope of such results. Finally, in order to maintain research rigor we have used a standardized interview method and topic design. That provided a solid foundation on which to frame our results. However, it also limited the scope of the thematic exploration. A new bespoke framework for exploring specifics in XR healthcare participatory design would be able to go further in depth and in detail of the topic. However, even maintaining these limitations in mind, the results of this work do, in fact, remain relevant.

Experiential healthcare education is becoming an essential part of contemporary curricula. Basic curricular topics such as anatomy and advanced manual skills, like surgery, benefit from the immediacy and multisensory engagement of XR content (37). With the emerging mainstreaming of XR healthcare education, the problem of content availability is becoming increasingly exacerbated (38) Co-creative healthcare XR content design emerges as a viable solution for covering the curricular needs of the modern healthcare classroom (39). Moving from the cumbersome, months long, development cycle of commercial resources participatory design has demonstrated the capacity to produce high quality resources on par with any currently implemented technology resource in healthcare education (40). However, designing the resources is only part of the challenge. The implementation and curricular integration in a valid and pedagogically acceptable way are important steps that become technical or administrative barriers, respectively, for XR healthcare teaching. Experiences, as they emerge from systematic pursuit of such endeavors demonstrate that there are tools that can streamline the technical implementation, providing semantic annotation, discoverability and accessibility of basic assets for content creation (41). Additionally, rigorous processes for proving the efficacy of co-creative content creation have been documented, allowing for the administrative and curricular acceptance of XR resources developed more easily (42).

In that context, the results of this thematic analysis shown that in order to achieve this level of acceptance for co-created XR educational resources and episodes two are the crucial steps as they we have identified in this work. The first step is the development of a bespoke methodological framework for XR educational content creation. XR content is significantly more development intensive and less granular, in their interactivity context, than other resources like virtual scenarios and video-casts. Adapting existing frameworks, to cater bespokely to generalized use cases for XR content development, could further increase efficacy of co-creative content development.

Pairing with the previous methodological step, an authoring environment that can quickly prototype XR resource concepts appears a natural complement to the previous methodological enabler. Preliminary tools for such an environment have been proposed (43) but wider availability is still forthcoming.

Concluding, XR educational resource development through participatory methods emerges as a promising approach but only when certain pre-requisites are met. Further work, both in the methodological frameworks and in the technical enablers is needed in order to successfully traverse the minefield of opportunities, which is XR educational content co-creation in healthcare.

## Data Availability

The raw data supporting the conclusions of this article will be made available by the authors, without undue reservation.
